# Leveraging Artificial Intelligence and Big Data to Optimize COVID-19 Clinical Public Health and Vaccination Roll-Out Strategies in Africa

**DOI:** 10.3390/ijerph18157890

**Published:** 2021-07-26

**Authors:** Bruce Mellado, Jianhong Wu, Jude Dzevela Kong, Nicola Luigi Bragazzi, Ali Asgary, Mary Kawonga, Nalamotse Choma, Kentaro Hayasi, Benjamin Lieberman, Thuso Mathaha, Mduduzi Mbada, Xifeng Ruan, Finn Stevenson, James Orbinski

**Affiliations:** 1 School of Physics, Institute for Collider Particle Physics, University of the Witwatersrand, Johannesburg 2050, South Africa; Bruce.Mellado.Garcia@cern.ch (B.M.); nalamotse.joshua.choma@cern.ch (N.C.); 716034@students.wits.ac.za (B.L.); 1144845@students.wits.ac.za (T.M.); Xifeng.Ruan@wits.ac.za (X.R.); finn.david.stevenson@cern.ch (F.S.); 2 iThemba LABS, National Research Foundation, Old Faure Road, Faure 7129, South Africa; 3 Centre for Disease Modelling, York University, Toronto, ON M3J 1P3, Canada; wujhhida@gmail.com (J.W.); jdkong@yorku.ca (J.D.K.); 4 Disaster & Emergency Management, School of Administrative Studies and Advanced Disaster, Emergency and Rapid-Response Simulation (ADERSIM), York University, Toronto, ON M3J 1P3, Canada; asgary@yorku.ca; 5 Gauteng Department of Health, Johannesburg 2107, South Africa; Mary.Kawonga@wits.ac.za; 6 School of Computer Science and Applied Mathematics, University of the Witwatersrand, Johannesburg 2050, South Africa; Kentaro.Hayashi@students.wits.ac.za; 7 Head of Policy at Gauteng Office of the Premier, Johannesburg 2107, South Africa; Mduduzi.Mbada@gauteng.gov.za; 8 Dahdaleh Institute for Global Health Research, York University, Toronto, ON M3J 1P3, Canada; orbinski@yorku.ca

**Keywords:** COVID-19, Africa, vaccine roll-out, big data, artificial intelligence

## Abstract

COVID-19 is imposing massive health, social and economic costs. While many developed countries have started vaccinating, most African nations are waiting for vaccine stocks to be allocated and are using clinical public health (CPH) strategies to control the pandemic. The emergence of variants of concern (VOC), unequal access to the vaccine supply and locally specific logistical and vaccine delivery parameters, add complexity to national CPH strategies and amplify the urgent need for effective CPH policies. Big data and artificial intelligence machine learning techniques and collaborations can be instrumental in an accurate, timely, locally nuanced analysis of multiple data sources to inform CPH decision-making, vaccination strategies and their staged roll-out. The Africa-Canada Artificial Intelligence and Data Innovation Consortium (ACADIC) has been established to develop and employ machine learning techniques to design CPH strategies in Africa, which requires ongoing collaboration, testing and development to maximize the equity and effectiveness of COVID-19-related CPH interventions.

**What is already known about this subject?** The still ongoing COVID-19 pandemic is generating massive health, social and economic costs, amplifying existing inequalities and disparities and creating new ones.

**What are the new findings?** Big data and Artificial Intelligence machine learning techniques and collaborations can be instrumental in an accurate, timely, locally nuanced analysis of multiple data sources to inform clinical public health decision-making related to COVID-19, vaccination strategies and their staged roll-out.

**What are the recommendations for policy and practice?** Ongoing collaborations between African countries and other countries, such as Canada, relying on big data and Artificial Intelligence machine learning techniques, can enhance testing and development to maximize the equity and effectiveness of COVID-19 related clinical public health interventions.

## 1. COVID-19 Vaccination Strategy as Part of Clinical Public Health Strategy and Interventions

The COVID-19 pandemic is a global public health emergency with immediate and secondary health, social and economic impacts, as well as cascading and compounding crisis effects that are global in nature, and that cannot be solved by any one country acting alone. More than 190 million people have been infected, more than four million people have died, and SARS-CoV-2 and its variants of concern (VOCs) continue to spread globally with differing morbidity and mortality patterns nationally and regionally [1,2]. While there is vast experience and expertise in Africa in fighting against epidemics and outbreaks, the ongoing and evolving COVID-19 pandemic both reveals vulnerabilities and presents opportunities for innovation across the continent [3]. This COVID-19 pandemic coincides with a rapidly digitizing economy and improvements of public health surveillance systems, creating rich data sources to power big data and Artificial Intelligence (AI) applications. While the virus itself and policy responses to it have illuminated and exacerbated pre-existing vulnerabilities and inequities, as with other governments worldwide, African governments have used non-pharmaceutical and public health measures to contain, control and mitigate the national impacts of the COVID-19 pandemic.

These measures include hand washing, use of masks, physical distancing, case finding, testing, contact tracing, isolation, quarantine, border controls, and restricted movement and gatherings [4]. While these interventions have contributed to saving lives, their secondary effects have imposed massive societal and economic burdens (including wide-spread job losses, reduced GDP, food insecurity), as well as massive disruptions in health services (for example in surgical care, maternal-child health, and national immunization programs) with concomitant increases in associated morbidity and mortality [5,6,7].

With unprecedented and ongoing clinical public health and scientific global collaboration in the year since the emergence of the COVID-19 pandemic, some new vaccines have been approved and are now being used under emergency use provisions by some nations and are pending emergency use approvals in many other nations. At least 230 other vaccine candidates are in varying stages of preclinical and clinical development, and some of these will likely be available for national emergency use approvals [8].

While many economically developed countries have already started immunizing, securing for themselves more than half of the world’s doses, most African nations are still waiting for vaccine stocks while preparing their vaccination campaigns. At the time of writing, a global average of 1.89 per 100 people has been vaccinated, while the continent of Africa is in last place with the number vaccinated at 0.04 per 100 people [9]. Driven by multiple bilateral advance purchase agreements, vaccine nationalism by rich nations shapes vaccine scarcity in poor nations, which means that three-quarters of all vaccines administered globally have occurred in only 10 countries, most of which are high-income countries [10]. Most African nations are relying mainly on the COVAX facility (a global collaborative co-financing vaccine procurement mechanism established to support equitable access COVID-19 vaccines) to allocate and then support at least a partial supply of effective vaccines.

## 2. Vaccine Nationalism and Impacts on Vaccine Scarcity

Deploying and administering effective and safe vaccines against COVID-19 to large populations at a fast pace is, indeed, a nontrivial task, given the factors above, the high transmissibility of the virus and its speed of spread, the variable feasibility of public health mitigation and prevention strategies, the infrastructure and logistics requirements, including cold chain, supply, distribution and delivery related issues, real-time monitoring and determination of vaccine efficacy, and vaccine production, as well as vaccine hesitancy, which is particularly high in the African continent [11,12].

Due to the logistical challenges in Africa, an assessment of different vaccination scenarios and roll-out strategies, that take into account national and local realities, is of paramount importance for public health policy and decision making, in order to maximize and track staged progress towards the achievement of herd immunity. The Africa-Canada Artificial Intelligence and Data Innovation Consortium (ACADIC) has been leveraging big data and AI-based techniques to respond to these policy questions in nine African countries.

## 3. The Africa-Canada Artificial Intelligence and Data Innovation Consortium (ACADIC)

The COVID-19 pandemic has been and is a catalyst for innovation, profoundly modifying the way vaccine products are conceived, manufactured, tested, and delivered. Many unprecedented collaborative innovation efforts to inform vaccination strategies at the global level are underway, which rely on recent scientific advancements, including big data and Artificial Intelligence (AI)-based techniques [13]. In particular, our Africa-Canada Artificial Intelligence and Data Innovation Consortium (ACADIC) brings together an interdisciplinary team of clinical public health experts and epidemiologists, physicists, statisticians, mathematicians, modelers, software engineers and data scientists from South Africa, Nigeria, Cameroon, Rwanda, Namibia, Botswana, Zimbabwe, Mozambique, Eswatini and Canada. We are developing and employing machine learning models to understand the impacts of clinical public health interventions.

ACADIC is also assisting with the design of vaccination strategies to help keep the number of COVID-19 cases requiring hospital care (including general ward hospital admission, high-level care and intensive care units or ICUs) below the maximum hospital capacity and maximize the impacts of available supply of vaccines, given the limited dose of vaccines that Africa may receive. We are building on the experience we have gained from South Africa, where ACADIC has designed and is piloting Artificial Intelligence-based techniques to model prioritization strategies in the COVID-19 vaccine roll-out in the Gauteng Province, the most populous of the nine provinces in South Africa.

In South Africa the national government is responsible for COVID-19 vaccine procurement and distribution to the nine provinces where provincial governments are responsible for storage, distribution to designated vaccination sites, and administration of vaccine to the population. An ACADIC team member is part of the Gauteng province premier’s COVID-19 Advisory Committee and is leading the COVID-19 related modelling initiatives in the committee.

## 4. The Smart Algorithm for Vaccine Optimization

In order to identify and prioritize target groups who can benefit most from vaccination, in the Gauteng Province a pilot study has been conducted, multi-dimensional data have been provided by the Gauteng Department of Health (GDOH) and are being used to characterize variables associated with severe illness. Fourteen dimensions of individual-specific characteristics have been modelled and include: age; eleven relevant co-morbidities; gender and ethnicity; information pertaining to the type of hospitalization (general ward, ICU, high-level care, and isolation) and whether the subject was discharged alive or died in care is also provided. This allows for implementation of a supervised machine-learning classification procedure. Multi-dimensional survey data are also available and provide representative data samples with a large number of inputs pertaining to social vulnerabilities. A subset of these inputs accords with the data inputs from the GDOH and matching between the two data sets has been performed. The data sets from the GDOH contain co-morbidities as an index of vulnerability that covers co-morbidities relevant to COVID-19. These include diabetes, hypertension, emphysema, bronchitis, asthma, pneumonia, heart disease, stroke, HIV/AIDS, and tuberculosis. The data sets provided by the GDOH contain about 5000 points of complete patient records for the period of the first wave of COVID-19 and the beginning of the second, where the remainder of the data are becoming available. This limited data set is sufficient to train smart algorithms with relatively high efficiency.

**Figure 1:**
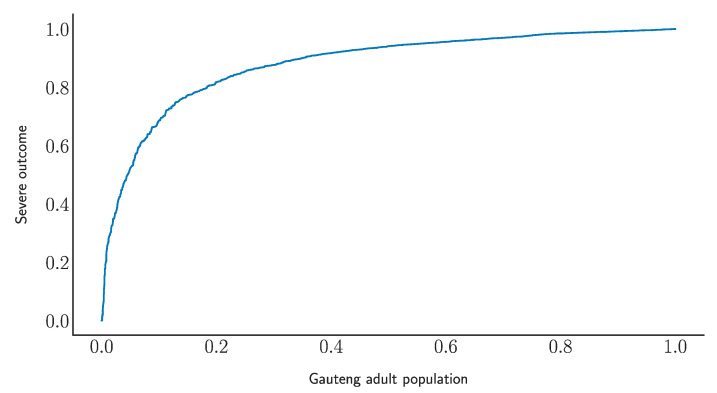
The receiver operating characteristic curve of the DNN model (see text).

A deep neural network (DNN) has been trained to separate two classes of data sets: severe illness (ICU, high-level care, and mortality) and less severe illness (subject discharged from the general ward). A training sample with 70% of the data has been used, where a testing sample with 30% of the data has been used to validate results and with which to estimate potential over-training. The corresponding DNN weights have been applied to the population data provided by the Gauteng City-Region Observatory [14]. Figure 1 displays the receiver operating characteristic curve of the DNN model. The horizontal axis shows the fraction of the adult population in Gauteng, where the vertical axis displays the reduction in severe illness. The DNN model indicates that by vaccinating about 20% of the adult population the probability of severe COVID-19 illness can be reduced by over 80%. Further refinements through multi-class labelling and enhanced data sets can improve the efficiency of the smart algorithm.

The DNN output provides a dimensionless number between 0 (highest risk of severe illness) and 1 (lowest risk of severe illness). Ideally, the population is classified according to the DNN output, which can be easily achieved through an electronic system. Further, AI-derived recommendations can be elaborated, as in Table 1, for classifying the risk of severe illness (1–greatest, 5–lowest risk) by demographic interval and by co-morbidity.

## 5. Variants of Concern (VOCs)

Several highly transmissible COVID-19 variants of concern (VOCs)—such as the South Africa VOC B.1.351, or the United Kingdom VOC B.1.1.7—have emerged and have spread internationally, with variable virulence and antigenicity relative to immune responses to infection with the resident strain, and relative to currently available vaccines.

The genomic characteristics and human immune response to resident and VOC strains of SARS CoV-2 must be matched with the antigenicity of currently available and soon to be available vaccines, and this data must be incorporated into big data AI applications. For both resident and VOC strains of the SARS-CoV-2 virus, big data AI applications can aid in the targeted identification and analysis of emergent hotspots or outbreaks; in the strategic choices for rapid, highly targeted and staged delivery of effective vaccines, as well as in ongoing monitoring for the efficient delivery and effectiveness of currently available vaccines and for vaccines that may become available in the future.

Since the required quantities of vaccines to reach herd immunity at national levels will not be immediately available to Africa, national allocation plans, and prioritization-based strategies are urgently needed to maximize clinical public health effectiveness and equity. To be effective, these strategies and plans must be locally informed and evidence-based and ensure staging and roll-out that achieves optimal health and health system and broader social and economic benefits with a gradually increasing supply of vaccines.

For instance, heterogeneous responses to the spread of COVID-19 are driven by a variety of factors, notably individual-specific characteristics (for example, age, gender, co-morbidity conditions, health, etc.), and characteristics specific to an individual’s behaviors and routine (e.g., geo-spatial, place of work, contact network and patterns of contacts). These factors can contribute to the emergence of epidemiological hot spots and understanding these, and their geo-locations is critical to deciding on how to most effectively and equitably prioritize the staged distribution of vaccines to critical demographic or vulnerable population categories (for instance, healthcare workers, vulnerable and frail elderly, etc.). Prioritization-based strategies must also be informed to address local obstacles and challenges, including that an adequate number of doses are procured and equitably distributed, according to a sustainable supply-chain output based on manufacturing and logistics capacity.

Indeed, a convergence of these issues has led to the cancellation in South Africa of the use of the Oxford AstraZeneca vaccine because of poor efficacy in reducing mild disease from the South Africa VOC B.1.351 [16,17]. However, using AI-derived evidence and analysis for vaccine prioritization we can expect the efficacy of the AstraZeneca vaccine to be significant in reducing severe COVID-19 disease (See Table 1).

## 6. Expanding AI Clinical Public Health Tools in Other Countries of Africa

Whereas more detailed data are available for South Africa (especially for the province of Gauteng), the ACADIC Consortium is developing networks and methodologies to transfer this pilot study, the knowledge gained and developed modelling techniques, to other African countries in a manner that accounts for differences in demographics and prevalence of co-morbidities and re-weighting the DNN algorithm initially developed for South Africa. We have already done this with Botswana, and we are doing this for seven other African countries. Validation and further correcting procedures will be implemented in order to ensure that the assumptions of the model are consistent with the specific features of the selected country. We are also specifically seeking to expand understanding of social vulnerabilities, which are paramount to model development and interpretation of the data. A range of sources—such as detailed geo-spatial maps of social vulnerabilities—will be utilized as inputs to our models. Further, gender is a major variable impacting COVID-19 in terms of risk of developing the communicable disease, disease severity, response to treatments, adverse reactions to medications and magnified social vulnerability [18] and is being actively incorporated across all AI models. We are also developing techniques that can help public health policy- and decision-makers devise enhanced COVID-19 testing policies and mass vaccination strategies [19,20,21] as well as assisting in identifying some coronavirus variants of concern (VOCs) that may evade immune responses triggered by vaccines and previous infections and/or rapidly responding to this possibility.

## 7. Conclusions

Big data and AI machine learning techniques, tools and collaborations can be highly instrumental in maximizing the equity and effectiveness of COVID-19 related CPH interventions. COVID-19 is a global public health emergency and has catalyzed international collaboration that brings diversified expertise to integrate big data and Artificial Intelligence-based techniques to inform clinical public health containment, control, and mitigation strategies in a rapidly evolving global pandemic, including optimal allocation of resources such as vaccines when global supply is unequally distributed. As manufacturing capacity is aligned with global demand for approved vaccines, national staged vaccine access allocation plans, and prioritization-based strategies are needed. Vaccine scarcity and the emergence of VOCs mandate novel decision support tools to develop vaccination priority and roll-out strategies to maintain critical public health and frontline service capacity, reduce the hospitalization and mortality and safely reopen essential business, and the economy. In conclusion, big data and AI machine learning techniques, tools and collaborations can be highly instrumental in a more accurate, timely, locally nuanced analysis of multiple sources of changing data to inform clinical public health decision making, including vaccination strategies and their staged roll-out, social vulnerabilities, gender, and strategies to identify and respond to VOCs.

## Figures and Tables

**Table 1:** Vaccine target population classification based on risk, as derived from the smart algorithm. Source: Gauteng Office of the Premier [15].

Risk Group	Age (Years)	Co-Morbidities	Population over 18 (%)
1	>60	Hypertension, diabetes, cardiac disease	3.8
2	50–60	Hypertension, diabetes, cardiac disease	3.7
3	40–50	Hypertension, diabetes, cardiac disease	3.7
4	>18	Any co-morbidity	18
5	>18	-	71

## Data Availability

Not applicable. All data are contained in the present editorial and related referenced publications.
